# Early Detection of the Initiation of Sit-to-Stand Posture Transitions Using Orthosis-Mounted Sensors

**DOI:** 10.3390/s17122712

**Published:** 2017-11-23

**Authors:** Abul Doulah, Xiangrong Shen, Edward Sazonov

**Affiliations:** 1 Department of Electrical and Computer Engineering, The University of Alabama, Tuscaloosa, AL 35487, USA; sayeed.doulah@gmail.com; 2 Department of Mechanical Engineering, The University of Alabama, Tuscaloosa, AL 35487, USA; xshen@eng.ua.edu

**Keywords:** orthosis, extreme learning machine, physical activity, posture recognition, sit-to-stand transition

## Abstract

Assistance during sit-to-stand (SiSt) transitions for frail elderly may be provided by powered orthotic devices. The control of the powered orthosis may be performed by the means of electromyography (EMG), which requires direct contact of measurement electrodes to the skin. The purpose of this study was to determine if a non-EMG-based method that uses inertial sensors placed at different positions on the orthosis, and a lightweight pattern recognition algorithm may accurately identify SiSt transitions without false positives. A novel method is proposed to eliminate false positives based on a two-stage design: stage one detects the sitting posture; stage two recognizes the initiation of a SiSt transition from a sitting position. The method was validated using data from 10 participants who performed 34 different activities and posture transitions. Features were obtained from the sensor signals and then combined into lagged epochs. A reduced number of features was selected using a minimum-redundancy-maximum-relevance (mRMR) algorithm and forward feature selection. To obtain a recognition model with low computational complexity, we compared the use of an extreme learning machine (ELM) and multilayer perceptron (MLP) for both stages of the recognition algorithm. Both classifiers were able to accurately identify all posture transitions with no false positives. The average detection time was 0.19 ± 0.33 s for ELM and 0.13 ± 0.32 s for MLP. The MLP classifier exhibited less time complexity in the recognition phase compared to ELM. However, the ELM classifier presented lower computational demands in the training phase. Results demonstrated that the proposed algorithm could potentially be adopted to control a powered orthosis.

## 1. Introduction

Sit to stand (SiSt) posture transition is a key component of mechanically demanding functional tasks in daily activities. The quality of life and functional independence highly depends on the ability to perform the SiSt posture transition. Many elderly people face difficulties in performing the SiSt transition successfully due to weakness in muscles. The failed transitions lead to a high occurrence of falls. In the US alone, each year, 2.5 million older people are admitted to emergency departments for fall injuries like head injury, broken bones and hip fracture [1]. Therefore, for the last two decades, many researchers have analyzed the detection and characterization of postural transitions, including the SiSt transition, in a variety of different powered orthoses. In particular, to provide movement assistance in SiSt transitions to elderly people, a potential solution is a lower limb orthosis, which is an external device/brace that can be attached to the lower part of the body.

With the advances in actuation technologies and high-performance materials (e.g., carbon fiber), compact and lightweight orthoses are becoming more common. Commercial powered lower-limb orthoses, such as AlterG Bionic Leg [2], are beginning to be used clinically for rehabilitation training. As such, it is reasonable to assume that powered lower-limb orthoses for older adults’ daily use would be available in the not-so-distant future. Recently, several orthoses have been developed for the treatment and rehabilitation of patients with lower-extremity problems [3,4,5], gait event detection [6,7,8] and assistance with locomotive activities of daily living [9,10,11,12,13,14,15]. Methods such as electromyography (EMG) [4,9,10,11,12,13,14,15,16,17], measurement of wearable sensors [6,18,19,20], and pressure platforms [21] have been used to recognize posture transitions.

Amongst all methods, surface EMG provides the most natural way to estimate the torque needed to perform a movement. An EMG interface to control a two-degrees-of-freedom orthosis to provide assistance for the shoulder joint while performing predefined movements was proposed in [9]. The studies of [4,11] proposed joint models for wearable powered orthosis with artificial pneumatic muscles at the ankle and the knee. The pneumatic muscles were controlled using surface EMG. The pattern of activation of leg muscles during SiSt transitions was reported by [16] to follow a predetermined order. A wearable robot Hybrid Assistive Limb was developed to provide physical support to patients in their daily tasks utilizing EMG signal [13]. To detect the intention of the sit-to-stand and stand-to-sit movements (StSi), features from different numbers of EMG channels (e.g., 2, 8 or 16 channels) were used with a range of classifiers (e.g., LDA, neuro-fuzzy, radial basis neural network) in [10,12,14,15,17].

Body-fixed sensors were introduced to movement analysis research in the early 1990s [16] and offered an alternative to EMG for the identification of SiSt transitions. The study referenced in [20] proposed a classifier (accuracy 96% in the healthy group and 92% in the chronic pain group) for SiSt and StSi detection in daily activity by placing a single inertial sensor on the trunk. A method using motion sensors to differentiate between the SiSt and the StSi transitions was presented in [21]. The authors observed that high-fall-risk elderly fallers experienced a lower and more variable transition duration. The accelerometers were also used in [22,23] to estimate the SiSt transition duration. The method proposed in [24] applied dynamic time warping to assess transitions during functional activities by gyroscope signals. The study of [25] investigated entropy as a metric to detect SiSt transitions using motion sensors. A sensor system containing a seismic accelerometer and gyroscopes was proposed in [26] to detect SiSt transitions. The study also investigated the transition duration and angular velocities to quantify SiSt transitions among young and older adults. An inertial sensor in [27] was located at the waist to analyze SiSt and StSi transitions. Recently, the authors in [28] proposed a method of detecting transitions (sensitivity 92% and specificity 88%) in Parkinson’s disease patients by placing a three dimensional accelerometer at the waist.

Getting ready for the standing-up motion is a process that is nontrivial for frail older adults. To reduce the load for the older users (both cognitive and physical), it is desirable to have an algorithm that automatically detects the user’s intention and initiates the motion assistance from the orthosis. While various methods have been proposed for the development and control of orthosis along with the detection of posture transition, significant challenges still exist for real-time implementation. In our previous work, we presented a multi-sensor based system to detect SiSt transitions without relying on complex EMG signals [29]. The recognition model was proposed using a support vector machine trained on features extracted from sensors placed on the orthosis. However, the algorithm presented in [29] resulted in a relatively low, but nevertheless significant amount of false positives. These false positives could force a seated person to receive unexpected motion which could lead to trauma or falls in the worst case scenario. Despite immense advances in the development of orthosis, less attention has been given to the issue of false positives in the SiSt detection. Therefore, the specific contributions of the current work are (1) the elimination of false positives for the reliable operation of the orthosis, (2) the early detection of the initiation of the SiSt transition and (3) the development of a computationally lightweight algorithm for early detection. A novel two-stage recognition algorithm is proposed to eliminate false positives and provide early detection of the transition. Neural networks classifiers (extreme learning machine and multilayer perceptron) were explored to develop a lightweight and computationally efficient SiSt detection algorithm.

## 2. Methods

### 2.1. Sensor System

**Figure 1:**
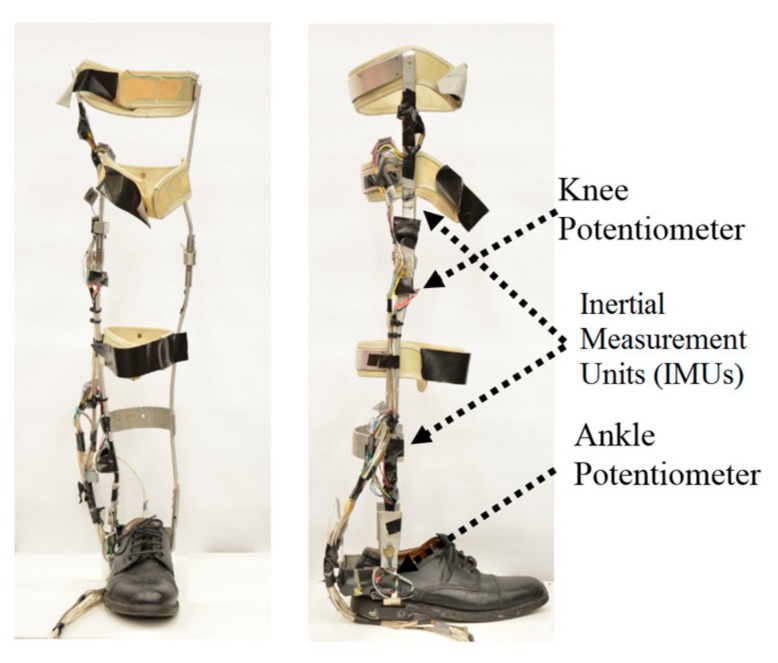
Sensors installed on the orthosis frame.

A metal-frame knee-ankle-foot orthosis [8] was instrumented with 14 sensors embedded in the frame of the orthosis. The chosen sensors were selected to monitor the kinematic segments and joints of the orthosis. Two inertial measurement units (IMUs; IDG500, Sparkfun Electronics, Niwot, CO, USA) each having a three dimensional accelerometer (ADXL335, Sparkfun Electronics, Niwot, CO, USA) and a two dimensional gyroscope (IDG500, Sparkfun Electronics, Niwot, CO, USA) were installed on the thigh and shank segments. The knee and ankle joints contained a rotary potentiometer to measure the angles. Force sensors in the shoe insole (Interlink Inc., Westlake Village, CA, USA) measured the contact force between the foot and ground. Figure 1 shows the sensors installed on the orthosis frame.

### 2.2. Data Collection Protocol

Ten healthy participants, 6 males and 4 females (mean age of 22 ± 3 years; mean height of 1.82 ± 0.02 m, mean body weight of 65.77 ± 10 kg) participated in the study. All the participants were confirmed to not have any motor impairments. Before the start of the experiment, the participants were informed of the purpose of the study and gave their written informed consent. The human study described herein was approved by the Institutional Review Board of the University of Alabama. The participants performed a set of 10 activities (Table 1) while wearing the orthosis on the right leg. The activities included posture transitions associated with daily activities such as sitting in different positions, standing, sit-to-stand and stand-to-sit transitions, and walking at slow, normal and fast speeds. To account for the variability of leg postures during sitting, all of the SiSt transitions were repeated with five different initial leg positions (Table 2). A data acquisition software (LJStream (LabJack Corporation, Lakewood, CO, USA) was utilized to record the data at the sample rate of 500 Hz. Each participant performed a total of 34 trials, with the duration of each trial being 60 s, resulting in a dataset of approximately 5.7 h of transition and activity data.

### 2.3. Signal Preprocessing

Before performing the annotation, the sensor signals were filtered using the translation-invariant wavelet transform to remove unwanted noise and then divided into consecutive non-overlapping frames of 100 samples (epochs) corresponding to 0.2 s of the sensor signal.

### 2.4. Data Annotation

**Figure 2:**
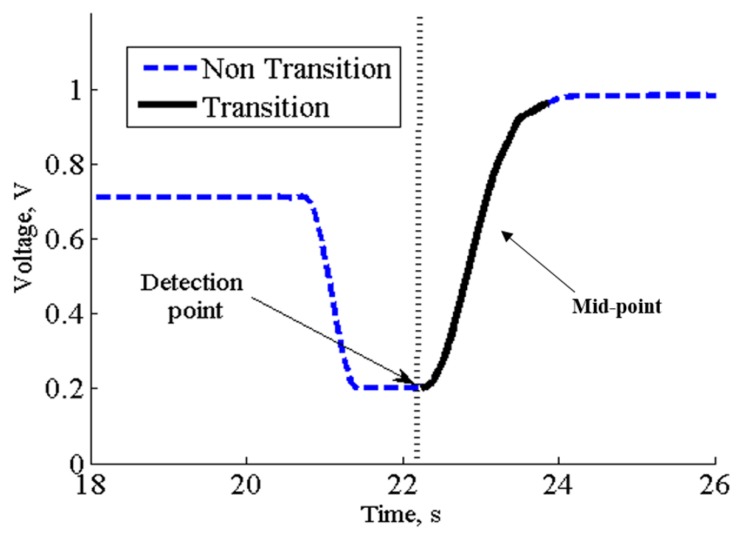
Signal waveform of knee potentiometer during a sit-to-stand activity; dashed and solid lines represent non-transition and the sit-to-stand transition period respectively.

The data annotation was performed to mark the SiSt transitions and the periods of sitting in the sensor signals. The signal from the knee potentiometer corresponding to 45° joint angle was used to label the midpoint of SiSt transitions (Figure 2). During the annotation of the SiSt transitions, all the data samples from the transition region were annotated as “1” and the samples outside of this region were annotated as “0”. Each 0.2 s epoch was labeled the same as the majority of signal samples in that epoch.

Periods of sitting were annotated in a similar manner. Epochs were labeled as “1” for the sitting posture and “0” for other activities. Since the annotation was carried out by three human raters, the reliability of manual annotation was assessed with Intra Class Correlation (ICC) coefficient. A high degree of agreement of 0.98 among the raters suggested a reliable annotation.

### 2.5. Feature Extraction

A set of 11 features was derived from each of the non-overlapping epochs (0.2 s time window) for each sensor signal. This time resolution was chosen using consideration of biomechanics of human movement [30]. The proposed resolution of 0.2 s is an order of magnitude less than a fast SiSt transition [30], therefore, guaranteeing that multiple epochs were present in any measured SiSt transition. The dimensionality of a feature vector describing an epoch was 154 (14 sensors × 11 features). Table 3 presents a list of features used. To eliminate potential dimensional inconsistencies, the feature vectors were normalized to the range [−1, 1] using Equation (1) where x represents features from original feature vectors and $x^{\prime }$ represents normalized features. The features from the neighbouring lagged epochs were concatenated to investigate the SiSt transition progression over time. Ten different numbers of lagged epochs (1 to 10) were tested.

(1) $$x^{\prime }=2\;\frac{x-\min x}{\max x-\min x}-1$$

### 2.6. Feature Selection: Minimum-Redundancy-Maximum-Relevance (mRMR)

The minimum Redundancy Maximum Relevance (mRMR) [31], which is a feature selection approach based on mutual information, followed by Forward Feature Selection (FFS) was used to reduce the number of features used in SiSt detection. The relevance in the mRMR method is described in terms of mutual information as in Equation (2). In the case of maximum relevance of a feature set S with m features {$x_{i},\;i=1,\ldots ,m$}, the selected features $x_{i}$ and target class c are necessary to determine the mutual information $I\left(x_{i};c\right)$. The largest mutual information implies the largest dependency on the target class. During a sequential search, the top m best individual features were then selected as Equation (3), based on the descent ordering of mutual information $I\left(x_{i};c\right)$.

(2) $$I\left(x_{i};c\right)=\iint p\left(x_{i},c\right)\log\frac{p\left(x_{i},c\right)}{p\left(x_{i}\right)p\left(c\right)}dx_{i}dc$$

where, $x_{i}$ and c are selected features and target class respectively and $p\left(x_{i}\right),\;p\left(c\right)$ and $p\left(x_{i},c\right)$ are their probabilistic density functions.

(3) $$\max Relv\left(S,c\right),\;Relv=\;\frac{1}{\left|S\right|}\underset{x_{i}\in S}{\sum }I\left(x_{i};c\right)$$

Two highly dependent features were considered redundant when they contribute the same class-discriminative power if any one of them was considered alone. The following Equation (4) was used to select mutually exclusive features with minimal redundancy condition.

(4) $$\min Redn\left(S\right),\;Redn=\;\frac{1}{{\left|S\right|}^{2}}\underset{x_{i},\;x_{j}\in S}{\sum }I\left(x_{i};x_{j}\right)$$

By measuring the relevancy and redundancy of computed features, a small set of significant features was achieved for both the continuous and discrete data sets. The mRMR can be either directly used or combined with other wrapper-based approaches as a two-stage feature selector. In order to achieve improved accuracy, the two-stage mode has been widely used for practical applications. Therefore, the present study used the two-stage approach, combining the mRMR with FFS.

### 2.7. Classifiers

Different classifiers perform differently, based on the application and data sets. In order to obtain a lightweight and computationally inexpensive classification algorithm, Artificial Neural Network (ANN) classifiers were explored. The ANN [32] is a biologically inspired computational model formed from artificial neurons that can optimize its performance by adjusting the weights of its neurons, based on the output errors. In this paper, two different types of ANNs were evaluated and compared.

#### 2.7.1. Extreme Learning Machine (ELM)

The extreme learning machine (ELM) [33] is a single-hidden layer feedforward ANN that offers a low training error and good generalization performance. For an extreme learning machine with n input neurons, L number of neurons on the hidden layer and N number of training cases trained on a feature set $\left(x_{i},\;t_{i}\right)$, the mathematical model can be represented as:

(5) $$\overset{L}{\underset{i=1}{\sum }}\beta_{i}g\left(a_{i}x_{i}+b_{i}\right)=\;o_{i}\;,\;j=1,\ldots ,\;N\;L\leq N$$

where $x_{i}={\left[x_{i1},x_{i2},\;\ldots ,\;x_{in}\right]}^{T}\;\in R^{n};$ is the input feature vectors, $t_{i}={\left[t_{i1},t_{i2},\;\ldots ,\;t_{im}\right]}^{T}\;\in \;R^{m};$ is the transition/non-transition labels; $\beta_{i}={\left[\beta_{i1},\beta_{i2},\;\ldots ,\;\beta_{iL}\right]}^{T}$ is the weight vectors of hidden to output layers; g(.) is the hidden layer activation function; $a_{i}={\left[a_{i1},a_{i2},\;\ldots ,\;a_{in}\right]}^{T}$ is the weight vectors of input to hidden layers; $b_{i}$ are the biases in the *i*th hidden layer neurons; and $o_{j}$ is the output of *j*th input training sample. When L=N, the above model can approximate all the training samples with zero error, $\sum_{j=1}^{N}∥o_{j}-t_{j}∥\;=0\;$ thus $\sum_{i=1}^{L}\beta_{i}g\left(a_{i}x_{j}+b_{i}\right)=\;t_{j}$

(6) Hβ=T

As our training sample was large, in order to reduce the computation complexity, the selection of L was less than N. In that case, the ELM assigned random parameters and calculated the output weights to the hidden nodes with a small error. The output weights were evaluated as:

(7) $$\beta =H^{+}T$$

where $H^{+}$ is the generalized inverse (Moore–Penrose) of the hidden layer output matrix H.

Because of the random assignment and the linear least squares estimation of weights, the training of the ELM is extremely fast. The number of hidden neurons varied for different epoch analysis. A cross validation procedure was performed to find out the optimal number of hidden neurons. The best number of hidden neurons was selected based on the lowest validation error and then carried out for classification.

#### 2.7.2. Multilayer Perceptron (MLP)

The multilayer perceptron (MLP) [34] is a class of feedforward ANN. The MLP neural network used in this work was comprised of three layers, namely (1) the input layer, where $x_{i}={\left[x_{i1},x_{i2},\;\ldots ,\;x_{in}\right]}^{T}\;\in R^{n}$ were the input feature vectors; (2) the output layer, where $Y={\left(Y_{1},\;Y_{2},\;\ldots ,\;Y_{n}\right)}^{T}\;\in R^{n}$ were the outputs; and (3) the hidden or intermediate layer, where $Z={\left(Z_{1},\;Z_{2},\;\ldots ,\;Z_{n}\right)}^{T}$ was the output of *q* neurons. The output of each neuron in the output and hidden layers can be represented by

(8) $$Z_{j}=f\left(\overset{n}{\underset{i=1}{\sum }}w_{ij}x_{i}-\;\theta_{j}\right)$$

(9) $$Y_{k}=f\left(\overset{q}{\underset{j=1}{\sum }}w_{kj}Z_{j}-\;\theta_{k}\right)$$

where f is a sigmoid transfer function; $w_{ij}$ is the weight; $\theta_{j}$ is the onset between hidden/input layers; and $\theta_{k}$ and $w_{kj}$ is the weight between the output/hidden layers. Similar to ELM, cross-validation was carried out to find out the optimal numbers of hidden neurons.

### 2.8. SiSt Transition Detection: Two Stage Recognition

**Figure 3:**
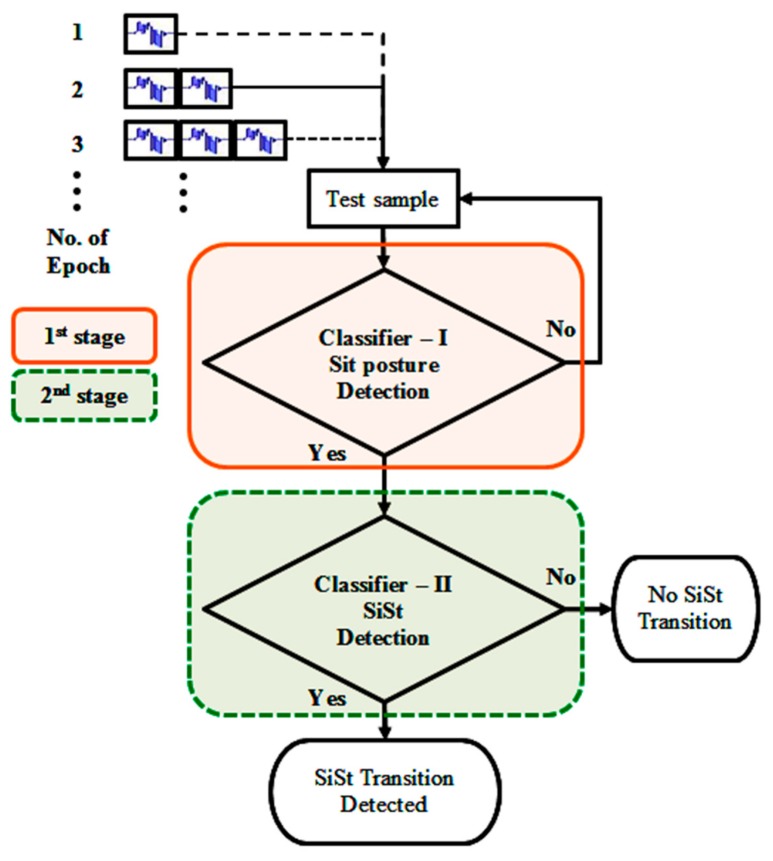
Flow diagram of the proposed two stage postural transition recognition.

Multistage hierarchical algorithms are well known in the field of machine learning and pattern recognition [34]. Previous related works [20,21,22,23,24,25,26,27,28] that primarily consider improving the accuracy of the transition detection did not, however, necessarily separate out the issue of false positives. In this paper, a new technique was proposed for both the detection of SiSt transitions and, especially, the elimination of false positives. The SiSt recognition utilized two stages of classification (Figure 3). In the first stage, the classifier detected candidate classes of “sitting posture” and in the second stage, the classifier determined whether a SiSt transition occurred or not.

The feature vectors and associated class labels were used to train the classifiers. For the purpose of training and validating the model, leave-one-out cross-validation was employed. In this procedure, the classifier was trained on all trials from 9 participants and tested on all trials from the remaining participant. This was repeated ten times for ten participants and the performance metrics were averaged across all validation results. All analysis were carried out in MATLAB (Mathworks Inc., Natick, MA, USA).

### 2.9. Performance Measures

To validate the ability of the proposed method to detect the initiation of a SiSt transition, the performance was evaluated on the epoch and transition levels. Additionally, to demonstrate the early detection of initiation, the detection time was computed for a different number of lagged epochs.

#### 2.9.1. Evaluation of Epoch-Level Detection

In the classification results, true positives (TP_e_) were defined as epochs correctly classified as SiSt transitions; true negatives (TN_e_) were defined as correctly classified “non-transition” epochs; the false positives (FP_e_) were defined as epochs incorrectly classified as SiSt transition epochs; the false negatives (FN_e_) were defined as SiSt transition epochs that were not recognized as such. The total FP_e_ was counted outside of the transition region.

True positive rate (TPR_e_), true negative rate (TNR_e_) and Accuracy_e_ as defined below were used to assess the accuracy of the classification on the epoch level.

(10) $${\mathrm{Accuracy}}_{e}=\;\frac{{\mathrm{TP}}_{e}+{\mathrm{TN}}_{e}}{{\mathrm{TP}}_{e}+{\mathrm{FP}}_{e}+{\mathrm{TN}}_{e}+{\mathrm{FN}}_{e}}\times 100\%$$

(11) $${\mathrm{TPR}}_{e}=\frac{{\mathrm{TP}}_{e}}{{\mathrm{TP}}_{e}+{\mathrm{FN}}_{e}}\times 100\%$$

(12) $${\mathrm{TNR}}_{e}=\frac{{\mathrm{TN}}_{e}}{{\mathrm{TN}}_{e}+{\mathrm{FP}}_{e}}\times 100\%$$

The F1_e_ score was derived as Equation (13).

(13) $$F1_{e}=\frac{2\times {\mathrm{TP}}_{e}}{2\times {\mathrm{TP}}_{e}+{\mathrm{FP}}_{e}+{\mathrm{FN}}_{e}}$$

#### 2.9.2. Evaluation of Transition-Level Detection

Due to the short epoch duration, a single SiSt transition typically corresponded to several transition epochs. For a particular trial, a single correctly detected transition epoch was sufficient to recognize the trial as a SiSt transition. The accuracy in detecting posture transitions in all the trials was evaluated as the number true positives (TP_t_), true negatives (TN_t_), false positives (FP_t_) and false negatives (FN_t_). In the classification results, TP_t_ was defined as the number of trials correctly classified as transition trials; TN_t_ was defined as the number of trials correctly classified as non-transition trials; FP_t_ was defined as the number of trials incorrectly classified as transition trials; and FN_t_ was identified as SiSt transitions that were not recognized and were defined as Failed to Detect (FTD).

#### 2.9.3. Detection Time (DT)

The detection time was defined as the time span between the first transition point labeled during the manual annotation and the first detection point labeled by the recognition model.

#### 2.9.4. Statistical Analysis

For both the ELM and MLP classifier-based SiSt recognition algorithms, the means of F1_e_ scores were calculated for different numbers of lagged epochs. Paired samples *t*-test was performed to the means, assuming equal variances to assess the sensitivity to change of the classifiers in the algorithm. A difference between means equals zero was used as a null hypothesis, and a non-zero difference was used as an alternative hypothesis. All analyses were done with Excel version 2013 (Microsoft Inc., Redmond, WA, USA), and a level of 0.05 was chosen for significance testing. Across all participants, a statistical *t*-test with 95% confidence was performed to compare mean F1_e_ scores obtained from two classification algorithms.

#### 2.9.5. Computational Complexity

In order to give an estimated time complexity, the execution time was evaluated in both training and recognition steps. All execution times were computed using MATLAB Profiler 2013 (The MathWorks, Inc., Natick, MA, USA).

## 3. Results

**Figure 4:**
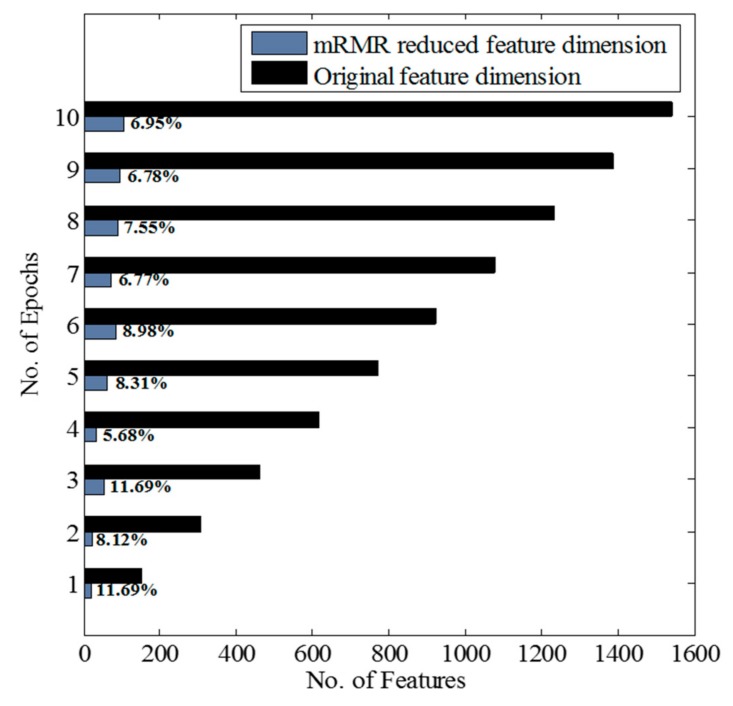
Feature dimension reduction using the mRMR algorithm.

**Figure 5:**
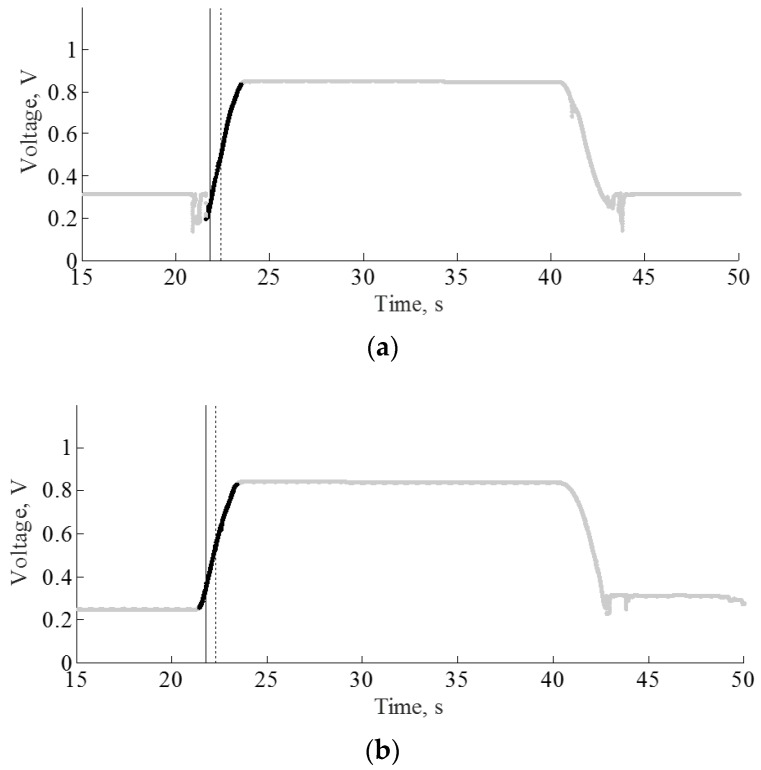
Activity mode of knee potentiometer of two different activities with different trials. Black solid lines represent detected transitions (**a**) activity-2 trial-6; (**b**) activity-6 trial-22.

**Figure 6:**
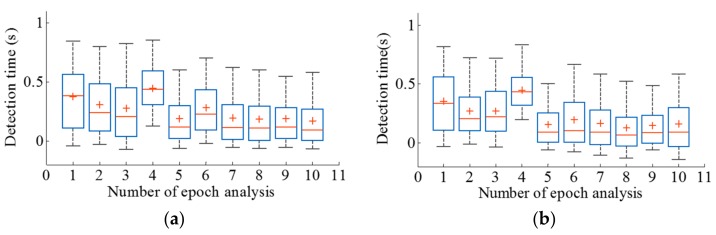
Box plots for detection times obtained from both algorithms: (**a**) ELM classifier; (**b**) MLP classifier.

After the feature computation, the application of the mRMR algorithm followed by FFS led the feature dimensions to be reduced to an average of 86% of their original size. Figure 4 illustrates the percentage of dimensionality reduction after the feature reduction process for different numbers of lagged epochs. The results of the assessment of the proposed method in terms of overall accuracy, detection time, false positives, and the number of the failure detection of SiSt transitions for both ELM and MLP classifiers are summarized in Table 4 and Table 5, respectively. Both ELM and MLP classifiers manifested the best performance in the case of the training feature vector for eight lagged epochs. The models presented 100% transition detection with no false positives at this lag. In transition level detection, both TP_t_ and TN_t_ were detected in 100% of the cases. Figure 5 illustrates the detection of SiSt transitions for two trials, employing the ELM-trained model. Both in Figure 5a,b, the model successfully detected the transitions without false positives. Figure 6 illustrates boxplots for the detection times obtained from both the classifiers.

The average duration of a SiSt transition in the collected dataset was 1.30 ± 0.55 s. On average, the proposed methods detected the initiation of SiSt posture transitions at 0.19 ± 0.33 s (ELM) and 0.13 ± 0.32 s (MLP) after the beginning of the transition. In terms of the percentage of total time spent on transition, the method detected the transition at 14.3% and 10.0% into the transition respectively.

**Figure 7:**
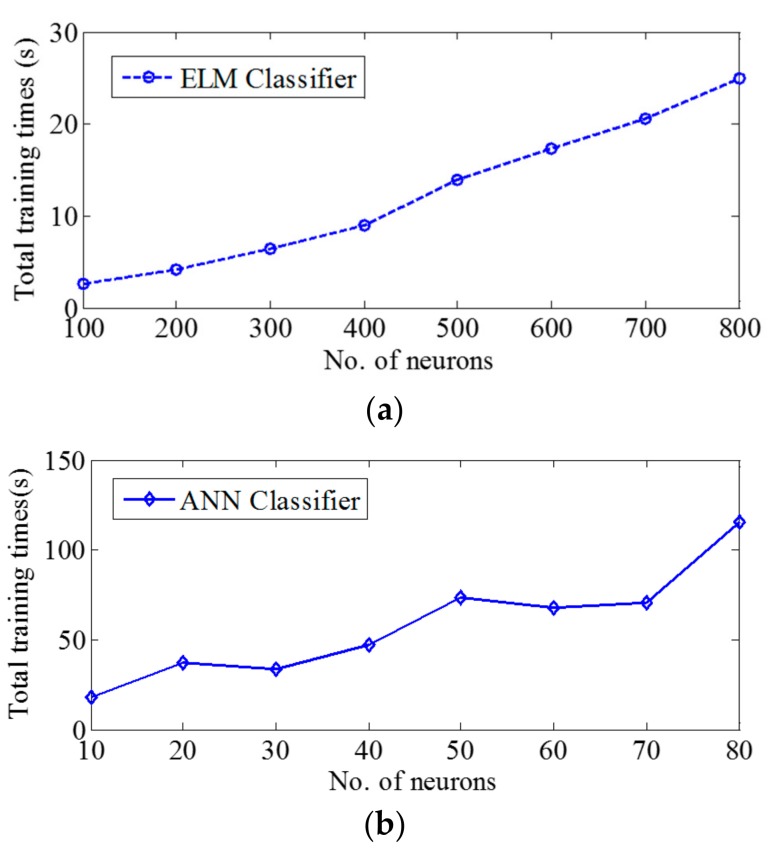
Total training times obtained from both classifiers: (**a**) ELM; (**b**) MLP.

Comparison of execution times of both classifiers is shown in Table 6. The MLP was faster at recognition than the ELM. Figure 7 shows the total training time for varying number of neurons. The total training time for the ELM model was found to be five times faster than for the MLP model. The statistical analyses were done in each epoch’s analysis. Following the best-performing number of epochs analyses, there was no significant difference found in F1_e_ scores obtained from the ELM and the MLP classifiers (*p* > 0.2027 and *p* > 0.0910 for seven and eight lagged epochs respectively).

## 4. Discussion

The main goal of this study was to develop of an algorithm that could provide a user the ability to perform SiSt transitions in a reliable and safe manner without false positives. In addition, a lightweight and computationally efficient algorithm was desired.

Contrary to the complex and burdensome EMG-based detection methods, the presented approach used simple sensors attached to the orthosis frame, rather than to the body. Therefore, the proposed method has a potential for enabling non-EMG orthosis that could offer reliable and consistent performance. The study referenced in [20] proposed a classifier for SiSt detection in daily activity and obtained 0.64 sensitivity and 0.82 specificity in the healthy group and 0.69 sensitivity and 0.74 specificity in the chronic pain group. Recently, the authors in [28] proposed a SiSt detection method in Parkinson’s disease patients with 0.92 sensitivity and 0.88 specificity. The specificity reported in these studies indicates a significant number of false positives. In the present study, the SiSt transitions were detected from a wide variety of initial postures without false positives. SiSt detection using the eight lagged epochs achieved 0.99 Accuracy_e_ and a 0.72 F1_e_ score in the case of the ELM classifier, and 0.99 Accuracy_e_ and 0.78 F1_e_ score in the case of the MLP classifier.

A range of 89–94% reduction in the size of the feature vectors was achieved utilizing the mRMR algorithm with FFS, substantially reducing the computational burden. Figure 4 illustrates the percentage of dimensionality reduction. The decrease in feature dimensionality could be potentially beneficial for real-time implementation of the algorithm on a microcontroller. Apart from the dimensionality reduction, it could be possible that not all of the sensors placed in the orthosis frame would contribute to the recognition of the SiSt transitions, which might allow the omission of some of the sensors in practical applications. Therefore, further investigation could be done to find out optimum location and selection of sensors.

One of the major contribution of the paper was the design of the two-stage recognition method. The proposed two-stage recognition method enabled the elimination of false positives in the detection of the SiSt transition. Note that every sit to stand transition state was associated with a sitting pattern. Therefore, during the recognition phase, the first stage classifier dealt with the detection of a sitting posture prior to the actual transition, which drastically reduced the possibility of incurring false positives. Given that the first stage classifier detected a sitting posture, the second stage classifier dealt with whether the SiSt transition had actually occurred or not. The system was able to eliminate possible false positives that occurred during activities associated with stand to sit transitions and walking. To the best of our knowledge, this paper is the first to critically study the false positives and propose an algorithm for the detection and elimination of false positives.

Our previous experiment [29] showed that the support vector machine classifier could potentially be used to recognize SiSt transitions. However, it is computationally intensive and not well-suited for real-time implementation on battery powered embedded processors. The extreme learning machine model has gained popularity in Electroencephalography (EEG) classification, fall detection and activity recognition. This paper introduced ELM in SiSt transition detection. In addition to the ELM-based classification model, the MLP model was also explored and compared. Based on the experimental results in Table 4 and Table 5, both MLP and ELM worked equally well in terms of classification accuracy. Table 6 demonstrates the execution time of both classifiers in the case of the eight lagged epochs. With regard to recognition time, the MLP model yielded faster recognition performance. The number of hidden neurons used in the algorithm directly impacted the computation complexity. For the best case eight lagged epochs, the number of neurons used in the MLP model (70) was much fewer than in the ELM model (600), therefore MLP outperformed ELM in recognition time. In terms of training time, the ELM model was found to be faster than the MLP model. Figure 7 illustrates the cumulative overall training time for the number of neurons. The ELM model was found to be more linearly proportional to the number of neurons used compared to the MLP model. Given the fact that both classification models produced comparable results, and the ELM required less training time, the ELM classifier could be a good candidate especially in on-line adaptive systems that may retrain or update the classifier on-line.

The results in Table 4 and Table 5 also show that the number of lagged epochs had an impact on the number of false positives. Over all tested lagged epochs, the ELM classifier was found to be less prone to false positives as compared to the MLP classifier. Regarding the TPR_e_, both the classifiers displayed lower accuracy at one lagged epoch and improved as the number of lagged epochs increased. It was observed that the TNR_e_ and Accuracy_e_ showed excellent performance in the cases of all lagged epochs. One potential reason of exhibiting such values could be the imbalanced dataset where the majority of the data belonged to non-transition labels. Therefore, the F1_e_ score was reported as a measure that did not take the true negatives into account. Note that the F1_e_ scores for different numbers of lagged epochs were greater than the TPR_e_ because false positives, one of the contributing factors in the computation of the F1_e_ scores, were virtually absent in the results. Also note that since the computed values were averaged across multiple trials, the mean values of TPR_e_ do not directly translate to the mean values of the F1_e_ scores.

The detection time was reported as a percentage of the transition period duration. Both the ELM and MLP classification algorithms were comparable in detection times. In Figure 6, the detection times obtained from both classification models are represented as a box plot, with the mean shown as a plus sign, the median as a central thick line, and the 25th and 75th percentiles as a box. It was observed that the range of detection times varied with the number of lagged epochs.

For first few lagged epochs (up to 6), the detection times for both models exhibited a varying time range with a standard deviation of 0.08. However, as the number of lagged epochs increased, the models tended to exhibit less variation, with a standard deviation of 0.01.

As for the percentage of total transition time, the MLP demonstrated almost 5% earlier detection as compared to the ELM model.

A statistical two-tailed *t*-test (*p* > 0.05) for the *F*1*_e_* score (*p* > 0.2027 and *p* > 0.0910 for seven and eight lagged epochs respectively) indicated that the choice of the classifier did not affect the recognition performance.

In spite of the fact that the proposed method offers potential use in the assistance of elderly people, a limitation of this study is that only young healthy individuals were included, elderly people being excluded due to the potential fall risk from a pilot orthosis device. Consequently, the implemented algorithms were not verified on elderly people who may perform movement at a slow pace. Therefore, further studies are needed to test the performance of the proposed system on a wider population, including frail elderly individuals. Another limitation of this study is that the SiSt transitions of the participants were detected under the specific protocol. More studies are needed to carry out research outside of the laboratory and in a free-living environment that encompasses real-life, everyday activities. Finally, the real-time implementation of the proposed neural network-based algorithm on low-power embedded processors is a potential extension of this work.

## 5. Conclusions

In this paper, a two-stage method for the detection of posture transition in lower limb orthosis was proposed. The findings demonstrated that the method could potentially offer early detection of the initiation of sit-to-stand transitions. In order to obtain a non EMG-based orthosis system with a lightweight algorithm, a sensor system comprised of an accelerometer, gyroscope and IMUs, were utilized in combination with a computationally inexpensive ELM classifier-based algorithm. Data were collected from 10 participants in order to validate the proposed algorithm. Significant features were selected by applying the mRMR algorithm followed by FFS. The performance of the ELM classifier was compared with the MLP classifier. Experimental results suggested that high accuracy can be obtained in SiSt transition detection. In terms of execution times, MLP classifier-based algorithm provided significantly lower computational costs. Overall, the proposed method based on both of the classifiers exhibited reliable detection of SiSt transitions without false positives, earliness in detection times, and a high detection rate. These results indication that the method could potentially be used to provide assistance to frail elderly people. The real-time implementation of the system would allow for conducting the experiment on elderly people in a free-living environment.

## Figures and Tables

**Table 1:** Data collection protocol—activities list.

Activity	Description
1	Sit comfortably on a chair for 60 s
*2*	*20 s sit, 20 s stand, 20 s sit*
*3*	*20 s sit, short distance walking and stand for 20 s, 20 s sit*
*4*	*20 s sit, 20 s stand, short distance walking and sit for 20 s*
5	60 s walking on a treadmill at normal speed
*6*	*Repetition of activity 2 with fast transitions*
*7*	*Repetition of activity 3 with fast transitions and walking*
*8*	*20 s stand, short distance walking and sit for 20 s*
9	60 s walking on a treadmill at 2–3 mph
10	60 s walking on a treadmill at 3–4 mph

**Table 2:** Initial seated positions.

Position	Description
0	Fully extended legs
1	Legs bent under the chair
2	Knees bent at 90-degree angle
3	Crossed ankles (left over right ankle)
4	Leg crossed (left leg over right knee)

**Table 3:** Features extracted from sensor signals.

Feature No.	Description	Feature No.	Description
1	Standard deviation	7	Median
2	Entropy	8	Slope
3	Coefficient of variation	9	Maximum to root mean square (RMS ratio)
4	Mean	10	RMS to mean ratio
5	Maximum	11	Fractal dimension
6	Minimum		

**Table 4:** Evaluation of the ELM model.

Epoch	TPR_e_	TNR_e_	ACC_e_	F1_e_	FTD	DT, s	% into the Transition	No. of FP_e_
1	0.1211	0.9994	0.9861	0.1713	14	0.38 ± 0.40	29.3	16
2	0.3255	0.9997	0.9889	0.4327	5	0.32 ± 0.37	24.3	2
3	0.4019	0.9994	0.9898	0.5071	4	0.28 ± 0.37	21.6	4
4	0.2508	0.9987	0.9872	0.3280	10	0.44 ± 0.33	33.6	5
5	0.6553	0.9991	0.9932	0.7240	0	0.19 ± 0.33	14.3	1
6	0.5023	0.9994	0.9913	0.6111	0	0.27 ± 0.32	20.8	0
7	0.6658	0.9990	0.9933	0.7283	0	0.20 ± 0.33	15.1	0
8	0.6510	0.9990	0.9931	0.7187	0	0.19 ± 0.33	14.3	0
9	0.6739	0.9990	0.9933	0.7360	0	0.20 ± 0.32	15.1	1
10	0.6718	0.9989	0.9932	0.7311	1	0.17 ± 0.32	13.0	0

**Note:** Epoch: Number of Epochs; TPR_e_: True Positive Rate in epoch; TNR_e_: True Negative Rate in epoch; ACC_e_: Accuracy in epoch; F1_e_: F1 score in epoch; FTD: Failed to Detect in transition; DT: Detection Time; % into the Transition: Detection of transition as percentage of total transition time; FP_e_: False Positives in epoch.

**Table 5:** Evaluation of MLP model.

Epoch	TPR_e_	TNR_e_	ACC_e_	F1_e_	FTD	DT, s	% into the Transition	No. of FP_e_
1	0.1673	0.9995	0.9869	0.2227	5	0.40 ± 0.40	31.0	6
2	0.5254	0.9995	0.9918	0.6315	2	0.23 ± 0.35	17.6	1
3	0.5262	0.9994	0.9917	0.6299	3	0.25 ± 0.36	19.1	8
4	0.3083	0.9986	0.9882	0.4048	4	0.43 ± 0.34	32.8	8
5	0.7038	0.9988	0.9937	0.7547	1	0.17 ± 0.32	12.8	1
6	0.6952	0.9988	0.9937	0.7518	1	0.15 ± 0.31	11.9	4
7	0.7416	0.9985	0.9942	0.7727	1	0.15 ± 0.31	11.3	0
8	0.7323	0.9987	0.9941	0.7765	0	0.13 ± 0.32	10.0	0
9	0.7381	0.9986	0.9940	0.7752	0	0.15 ± 0.31	11.7	0
10	0.7370	0.9986	0.9941	0.7753	0	0.12 ± 0.30	9.2	2

**Table 6:** Classifier execution times.

Classifier	Number of Neurons Used	Training Time 1st Stage	Training Time 2nd Stage	Recognition Time 1st Stage	Recognition Time 2nd Stage
ELM	600	8.6570	8.7038	0.0410	0.0371
MLP	70	44.2000	23.4580	0.0110	0.0110
